# *KRAS* biomarker testing disparities in colorectal cancer patients in New Mexico

**DOI:** 10.1016/j.heliyon.2017.e00448

**Published:** 2017-11-21

**Authors:** Alissa Greenbaum, Charles Wiggins, Angela LW Meisner, Manuel Rojo, Anita Y Kinney, Ashwani Rajput

**Affiliations:** a Department of Surgery, University of New Mexico, Albuquerque, NM, United States; b New Mexico Tumor Registry, Department of Internal Medicine, University of New Mexico, Albuquerque, NM, United States; c Department of Internal Medicine, University of New Mexico, Albuquerque, NM, United States; d University of New Mexico Comprehensive Cancer Center, University of New Mexico, Albuquerque, NM, United States; e University of New Mexico School of Medicine, University of New Mexico, Albuquerque, NM, United States

**Keywords:** Oncology, Health sciences, Clinical genetics

## Abstract

**Introduction:**

American Society of Clinical Oncology (ASCO) guidelines recommend that all patients with metastatic colorectal cancer (mCRC) receive KRAS testing to guide anti-EGFR monoclonal antibody treatment. The aim of this study was to assess for disparities in *KRAS* testing and mutational status.

**Methods:**

The New Mexico Tumor Registry (NMTR), a population-based cancer registry participating in the National Cancer Institute’s Surveillance, Epidemiology and End Results program, was queried to identify all incident cases of CRC diagnosed among New Mexico residents from 2010 to 2013.

**Results:**

Six hundred thirty-seven patients were diagnosed with mCRC from 2010–2013. As expected, *KRAS* testing in Stage 4 patients presented the highest frequency (38.4%), though testing in stage 3 (8.5%), stage 2 (3.4%) and stage 1 (1.2%) was also observed. In those with metastatic disease, younger patients (≤ 64 years) were more likely to have had testing than patients 65 years and older (p < 0.0001). Patients residing in urban areas received *KRAS* testing more often than patients living in rural areas (p = 0.019). No significant racial/ethnic disparities were observed (p = 0.66). No significant differences were seen by year of testing.

**Conclusion:**

Age and geographic disparities exist in the rates of *KRAS* testing, while sex, race/ethnicity and the year tested were not significantly associated with testing. Further study is required to assess the reasons for these disparities and continued suboptimal adherence to current ASCO KRAS testing guidelines.

## 1 Introduction

Colorectal cancer (CRC) is the third leading cause of cancer deaths in the United States [1]. Nearly 20% of patients are found to have metastatic disease at the time of diagnosis [2]. The American Society of Clinical Oncology (ASCO) published guidelines in 2009 recommending all patients with metastatic colorectal cancer (mCRC) receive *KRAS* testing to guide anti-epidermal growth factor receptor (anti-EGFR) monoclonal antibody (MoAb) treatment [3]. Approximately 40–60% of colorectal cancers harbor a *KRAS* mutation [4, 5, 6]. This activating missense mutation was previously identified at codons 12 and 13, and most recently exons 3 and 4 [7]. Stage IV CRC patients with wild-type *KRAS* status demonstrate improved progression-free survival (PFS), objective response and overall survival (OS) after receiving chemotherapy in combination with anti-EGFR MoAb therapy, while those with *KRAS* mutations do not benefit from cetuximab or panitumumab [8, 9, 10, 11, 12, 13, 14].

Since the publication of the 2009 ASCO guidelines, recent literature has shown increased physician awareness of the need for *KRAS* testing and increased testing rates [15]. However, 50% or more of eligible patients do not receive the test [6, 16, 17, 18]. Various underserved populations such as rural residents, Hispanics, American Indians and elderly patients often have inferior oncologic outcomes and reduced access to cancer care [19, 20, 21, 22, 23]. It remains unclear if differences in genomic testing rates exist between sexes, ancestral groups, and place of residence. In 2010, New Mexico was found to have the highest rates of *KRAS* testing out of 18 registries in the National Cancer Institute’s (NCI) Surveillance, Epidemiology, and End Results (SEER) program [18]. New Mexico, a multi-cultural state with a large proportion of rural areas presents an ideal setting to assess disparities in *KRAS* testing.

This study aimed to assess for disparities in *KRAS* testing and mutational status in the state of New Mexico, as well as to characterize testing trends over time. We hypothesized that certain population subgroups with mCRC, including older patients, racial/ethnic minorities and rural residents would be less likely to receive guideline-based testing.

## 2 Materials and methods

### 2.1 Patient cohort and variables

This investigation was conducted with existing records from the population-based New Mexico Tumor Registry (NMTR), a founding member of the NCI’s SEER Program that has continuously participated in the SEER Program since 1973. Eligible cases were defined as New Mexico residents diagnosed with CRC (International Classification of Disease for Oncology-Third Edition (ICDO-3) anatomic site codes C18.0-C20.9 and C26.0) during calendar years 2010–2013. This time-period represents the first four full years that *KRAS* testing was documented in NMTR and in the SEER Program. The analysis was restricted to individuals with malignant CRC (ICDO-3 behavior code 3); individuals with benign and in-situ disease were excluded from the analysis. Cases of lymphoma (ICOD-3 histology codes 9590–9992), Kaposi Sarcoma (ICDO-3 histology code 9140) and mesothelioma (ICDO-3 histology codes 9050–9055) were also excluded from the analysis. Because of the relatively small number of CRC cases diagnosed among African Americans, Asians, and other racial/ethnic groups in New Mexico, this analysis was restricted to non-Hispanic Whites, Hispanics, and American Indians. Analysis of the predictors of KRAS testing was further restricted to individuals with AJCC Stage IV disease.

Performance of *KRAS* testing was documented from specific statements in medical records and was coded according to standards promulgated by the SEER Program. Individuals were considered to have been tested for *KRAS* if results were classified as “abnormal” or “normal”. If the medical record indicated that the test had been ordered, but the result was not documented in the chart, cases were classified as not having been tested for *KRAS*. The definitions of *KRAS* testing and results are consistent with those utilized in a previous analysis of SEER Program data [18].

Variation in KRAS testing was assessed by several patient characteristics, including age at diagnosis (<40, 40–64, and 65+ years), race/ethnicity (American Indian, Hispanic, and non-Hispanic white), calendar year of diagnosis (single years 2010–2013), sex (female, male), urban vs. rural place of residence at time of diagnosis (based on census-tract of residence) and per-capita income (quartiles, based on census tract of residence). This investigation was approved by the University of New Mexico Human Research Protections Office.

### 2.2 Statistical analysis

Receipt of *KRAS* testing was assessed for each of the above-listed patient characteristics with chi-squared tests and by univariable logistic regression. Multivariable logistic regression was used to assess receipt of *KRAS* test for each patient characteristic while simultaneously adjusting for all other characteristics. All statistical tests were two-sided and assessed at an alpha level of p < 0.05. All statistical analyses were conducted with standard modules of the Statistical Analysis System (Version 9.4, SAS Institute, Cary, NC).

## 3 Results

**Fig. 1:**
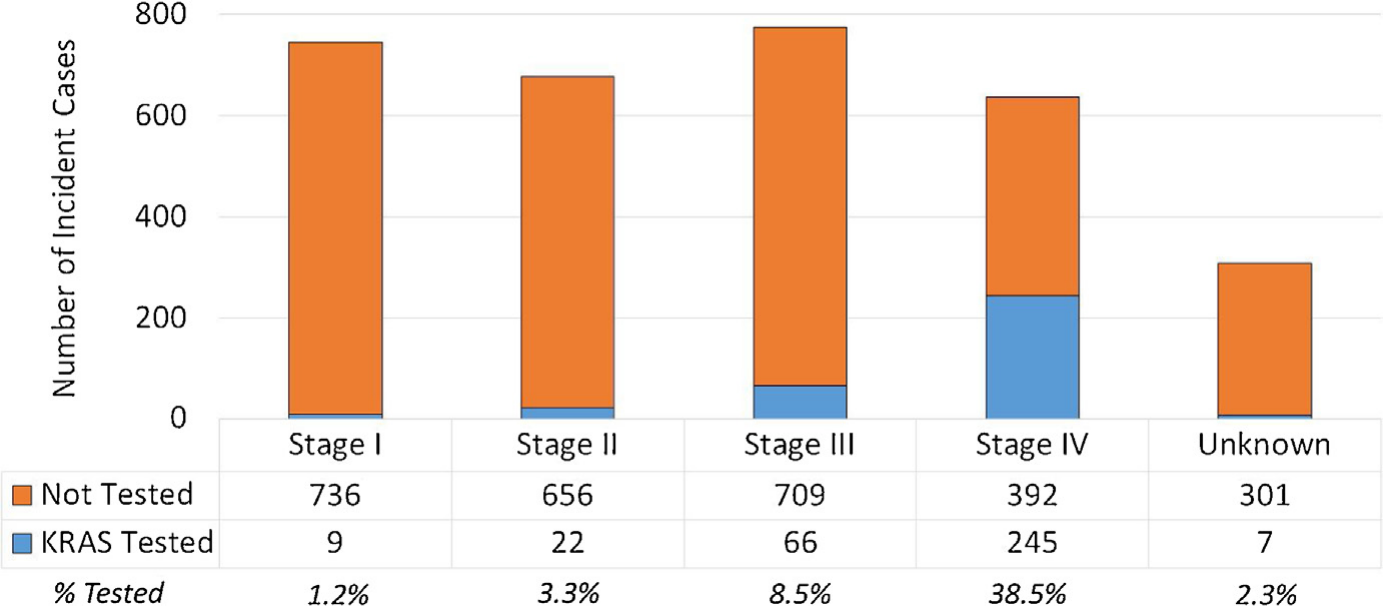
Frequency of *KRAS* testing by AJCC 7th edition staging, shown by number of incident cases and percentage. The majority of testing was performed in stage IV patients as expected, though a total of 13.1% of patients in stage I through III also received *KRAS* testing.

Overall, 3,142 patients were diagnosed with CRC in New Mexico from 2010 to 2013. Baseline patient and tumor characteristics are shown in Table 1. AJCC 7th edition [24] stage of disease at the time of diagnosis was distributed as follows: 23.7% (n = 743) were stage I, 21.6% (n = 678) were stage II, 24.7% (n = 775) stage III and 20.3% (n = 637) stage IV. Unknown stage constituted 9.8% (n = 308) of patients. As expected, *KRAS* testing was significantly higher in stage IV cases (38.5%; p = 0.0001), though guideline discordant testing was observed stage III (8.5%), stage II (3.2%) and stage I (1.2%) (Fig. 1).

**Table 1:** Selected characteristics of incident CRC cases diagnosed among adult residents of New Mexico during the time period 2010-2013.

Characteristic	Category	Number of Cases	Percent of Total
Ancestry	Non-Hispanic White	1,704	54.23
	Hispanic	1,117	35.55
	American Indian	218	6.94
	Other	103	3.28

Sex	Male	1,690	53.79
	Female	1,452	46.21

Age at Diagnosis	21-39 years	75	2.39
	40-64 years	1,295	41.22
	65+ years	1,772	56.40

Anatomic Sub-Site	Right Colon	1,167	37.14
	Left Colon	752	23.93
	Overlapping Regions of Colon	23	0.73
	Colon, Not Otherwise Specified	240	7.64
	Rectum & Recto-Sigmoid Junction	960	30.55

AJCC Stage	I	743	23.65
	II	678	21.58
	III	775	24.67
	IV	637	20.27
	Unknown	309	9.83

Surgery	Yes	2,304	73.33
	No	705	22.44
	Unknown	133	4.23

Radiation	Yes	435	13.84
	No	2,545	81.00
	Unknown	162	5.16

Place of Residence	Urban	2,030	64.61
	Rural	1,106	35.20
	Unknown	6	0.19

### 3.1 *KRAS* testing in stage IV patients

Table 2 summarizes rates of *KRAS* testing in stage IV non-Hispanic white, Hispanic, and American Indian CRC patients. After adjusting for age, sex, ancestry, area of residence and year of testing, younger patients (ages 22–39 and 40–64 years) were more likely to receive testing than patients 65 years and older (p < 0.0001). There were no testing disparities with regard to sex (p = 0.669) and ancestry (p = 0.378). Patients living in an urban area were more likely to receive *KRAS* testing than patients living in rural place of residence (41.8% vs. 31.9%, p = 0.017). A modest increase in *KRAS* testing rates among mCRC patients was observed from 2010 to 2013 (34.2% to 43.6%) though this was not statistically significant.

**Table 2:** Multivariable analysis of *KRAS* testing rates in Stage IV Colorectal Cancer.

	Number of cases, total	Tested for KRAS, n (%)	Adjusted** Odds Ratio 95% CI		P
Sex					
Male	331	130 (39.3)	1.00	Reference (Ref)	0.669
Female	282	106 (37.6)	0.88	0.63–1.24	
Age (years)					
22-39	19	12 (63.2)	1.00	Ref	<0.0001
40-64	284	141 (49.7)	0.62	0.23–1.63	
65+	310	83 (26.8)	0.22	0.05–0.59	
Ethnicity					
Non-Hispanic White	325	126 (38.8)	1.00	Ref	0.378
Hispanic	246	98 (39.8)	1.04	0.63–1.71	
American Indian	42	12 (28.6)	0.71	0.30–1.68	
Year of diagnosis					
2010	158	54 (34.2)	1.00	Ref	0.167
2011	155	53 (34.2)	1.00	0.61–1.61	
2012	160	68 (42.5)	1.42	0.88–2.27	
2013	140	61 (43.6)	1.41	0.87–2.28	
Place of residence					
Urban	409	171 (41.8)	1.00	Ref	0.017
Rural	204	65 (31.9)	0.67	0.45–0.98	
Annual income, per capita (census tract)					
$5,051 − $15,656	153	52 (34.0)	1.00	Ref	0.618
$15,662 − $23,034	153	62 (40.5)	1.48	0.88–2.48	
$23,126 − $32,042	153	60 (39.2)	1.27	0.68–2.35	
$32,138 − $84,620	152	61 (40.1)	1.21	0.63–2.34	

*The following characteristics had no missing values: age at diagnosis, ancestry, calendar year, sex and place of residence. Census tract data for per capita income was unavailable in two cases.

**Odds ratios adjusted for variables listed in table.

### 3.2 Difference in *KRAS* mutational status of stage IV patients

Of the stage IV CRC patients who received *KRAS* testing, 43.3% had a missense mutation. There were no significant differences in rates of wild-type versus mutant status when examined by age (p = 0.693), sex (p = 0.182), ancestry (p = 0.774), year of diagnosis (p = 0.628), per capita income (p = 0.870) or geographic area (p = 0.205) (Table 3).

**Table 3:** Comparison of KRAS mutation rates among patients tested.

	Number of cases, total	KRAS mutation, n (%)	Adjusted** Odds Ratio 95% CI		P
Sex					
Male	129	52 (40.3)	1.00	Reference (Ref)	0.182
Female	104	51 (49.0)	01.53	0.88–2.66	
Age (years)					
22-39	12	4 (33.3)	1.00	Ref	0.693
40-64	139	61 (43.9)	1.82	0.49–6.73	
65+	82	38 (46.3)	2.15	0.56–8.27	
Ethnicity					
Non-Hispanic White	125	55 (44.0)	1.00	Ref	0.774
Hispanic	97	42 (43.3)	0.71	0.33–1.51	
American Indian	11	6 (54.5)	1.47	0.33–6.61	
Year of diagnosis					
2010	54	26 (48.2)	1.00	Ref	0.628
2011	53	20 (37.7)	0.63	0.28–1.39	
2012	66	28 (42.4)	0.75	0.35–1.60	
2013	60	29 (48.3)	0.92	0.43–1.98	
Place of residence					
Urban	169	79 (46.8)	1.00	Ref	0.205
Rural	64	24 (37.5)	0.55	0.88–2.66	
Annual income, per capita (census tract)					
$5,051 − $15,656	51	25 (49.0)	1.00	Ref	0.870
$15,662 − $23,034	61	26 (42.6)	0.86	0.38–1.99	
$23,126 − $32,042	60	25 (41.7)	0.53	0.20–1.38	
$32,138 − $84,620	60	26 (43.3)	0.58	0.21–1.57	

## 4 Discussion

With the advent of precision oncology, genomic testing has become the standard of care for many types of cancer. *KRAS* was among the first biomarker tests that became widely used to guide cancer treatment. ASCO and the National Comprehensive Cancer Network (NCCN) both published clinical guidelines in 2009 recommending that patients with mCRC receive *KRAS* testing of their tumors to guide the delivery of anti-EGFR therapy [3, 25]. Recent studies have shown that less than half of patients with mCRC are tested according to these guidelines [18]. Disparities in the rates of *KRAS* testing were identified in this study, with older patients and those residing in rural areas being less likely to receive testing.

Overall the rate of *KRAS* mutation in our study was 43.3% which is comparable to previous literature [4, 5, 6]. Few studies have been dedicated to exploring possible disparities in *KRAS* mutations in CRC, though some retrospective data suggest mutations are more likely to be observed in elderly patients and Asian females compared to Asian males [26, 27]. We found no differences in *KRAS* mutation rates between sex, age, ancestry, geographic or income groups.

Our findings are comparable with recent studies showing that patients living in or near a metropolitan area are more likely to receive *KRAS* testing. An evolving shortage of oncology specialists, fewer primary care physicians, and longer travel times to a cancer center likely affect access to care of those living in rural areas [19, 28, 29, 30]. With nearly 20% of CRC patients nationally living in non-metropolitan areas (over 30% in our cohort), these disparities create substantial effects. Patients living in rural areas have increased mortality rates and risk of death in CRC [30, 31]. Increased distance to an academic center and rural residency are also associated with less frequent receipt of adjuvant chemotherapy [30, 32, 33]. Our data suggests that rural residency is not only associated with treatment and outcomes disparities, but also decreased genomic testing in mCRC patients.

This study also confirms age-related disparities in cancer care. Elderly patients often do not receive guideline-concordant care in CRC. The median age of CRC diagnosis in the U.S. is 69 years, making this topic especially significant [34]. Increased prevalence of medical comorbidities and organ dysfunction in the elderly are legitimate concerns regarding the initiation of cancer treatments [34]. Schiphorst et al. demonstrated that guideline adherence for stage I–III rectal cancer in the Netherlands decreased correspondingly with age [35]. In a 2010 case vignette survey, oncologists were less likely to start chemotherapy for a patient with newly diagnosed mCRC based on advanced age alone, regardless of performance status [36]. The initiation of therapy, either for curative or palliative intent, must be weighed with the risks of adverse effects from anti-EGFR moAb therapy. Patients require monitoring for hypomagnesemia, as well as skin and gastrointestinal toxicities [34]. However, active geriatric patients with comparable functional status to younger patients with mCRC should be equally considered for anti-EGFR treatment [34].

There are several potential explanations for the finding that patients with mCRC 65 years and older do not receive *KRAS* testing. It is true that a provider may not recommend testing due to poor performance status, comorbidities, and short life-expectancy, which are more prevalent in older patients. In our study, it is unclear whether *KRAS* testing is less likely to occur as the result of discussions between patient and provider, or if the test is simply not offered. It is possible that patients with metastatic disease may refuse treatments such as anti-EGFR therapy, rendering *KRAS* status of little clinical value. Concerns about the financial burden of *KRAS* testing may also play a role in decision-making. Anti-EGFR medications range from approximately $3000 to $6000 cost per dose and *KRAS* testing costs approximately $200, though both are covered by Medicare and Medicaid in New Mexico with few exceptions.

Current clinical practice guidelines indicate that *KRAS* testing should occur in mCRC patients to provide guidance on whether response to anti-EGFR therapy is expected [3, 25], though consistent adoption of *KRAS* testing remains poor. New Mexico exhibits high testing rates compared to the rest of the nation [18], though overall rates are still low, occurring in less than 40% of patients with mCRC. In contrast, a 2016 survey of 34 Kaiser Permanente oncology providers from seven centers [37] self-reported consistent ordering and rapid adoption of *KRAS* testing within 6 months of National Comprehensive Cancer Network (NCCN) guideline publication, though overall rates were not measured. While patient factors may contribute to receipt of *KRAS* testing, literature examining general adoption of cancer guidelines suggests the most important influence remains the ordering physician [38].

One source of testing inconsistency may relate to the fact that the two prominent organizations providing these guidelines have subtle differences in indications for *KRAS* testing. Per NCCN guidelines, all stage IV CRC patients should be tested, while ASCO recommends only for those being considered for anti-EGFR therapy should be offered the test. As utilizing this type of genetic analysis is a relatively new practice guideline, some oncology providers may be uncertain which guidelines to follow as well as how to interpret *KRAS* status results in order to make subsequent treatment recommendations. As research on genomic testing of tumors is generated at a fast pace, guidelines regarding testing in cancer are prone to constant modification. For example, it is now known that patients with mutations in exon 2 (codons 12 or 13) will derive no benefit from cetuximab or panitumumab and have poorer prognosis [25]. More recent ASCO guidelines have expanded to recommend tumor testing for mutations in *KRAS* exons 3 (codons 59 and 61) and 4 (codons 117 and 146) as findings from recent Phase II and III trials indicated patients with these mutations also will not benefit from anti-EGFR therapy [7]. While personalized medicine offers exciting new promise in cancer treatment, investment in education, clinical pathways prompting use, and accountability is imperative to successfully implement novel tests and clinical guidelines [38].

It is noteworthy that over one-quarter of patients tested in this cohort had non-metastatic disease, which is not recommended. While ASCO and NCCN both recommend testing of stage IV patients only, a small but developing body of literature advocates for the utility of *KRAS* testing in those with localized disease [39]. *KRAS* testing in patients with stage I through III disease is controversial and contrary to current clinical guidelines. Roth et al. reported no major prognostic value in stage II–III CRC in a multivariate analysis of 3,278 patients enrolled in an adjuvant trial [40]. Conversely, two recent studies conclude *KRAS* and *BRAF* mutations are associated with inferior survival in Japanese patients with stage I–III disease [27] and significantly poorer disease-free survival (DFS) (3-year DFS 79% and 92% in mutant and wild-type respectively; p = 0.006) [26]. Despite claims that *KRAS* status is an independent predictor of clinical outcome in resectable CRC, the aforementioned paper does not currently advocate for obtaining *KRAS* testing to guide prognosis discussions with patients. Thus, the clinical utility of obtaining *KRAS* testing in this patient population remains unclear. The high rates of testing in local and regional CRC observed in our study may stem from provider motivation to supply the patient with more information or from a misunderstanding about the current recommended use of the test. This underscores the need to further study reasons for over-testing and to guide future interventions to promote guideline-consistent care.

Our study has several limitations. The NMTR cannot ascertain whether *KRAS* testing was offered to a patient by a provider, only whether it was done or not. In regards to ancestry, American Indians were less likely to receive *KRAS* testing (28.6%) compared to non-Hispanic whites (38.8%) and Hispanics (39.8%) but this was not statistically significant. There were only 42 American Indians in our cohort, which limited statistical power. As American Indians compromise over 10% of the New Mexico population, a disparity in *KRAS* testing rates may be elucidated with more data in upcoming years.

## 5 Conclusion

Compared to national averages, New Mexico exhibits high rates of *KRAS* testing in patients with mCRC. Age and geographic testing disparities exist, while sex, ancestry and the year tested showed no significant differences. Further study is required to delineate reasons for these disparities in *KRAS* testing and survival outcomes, as well as to determine the motivation for testing in stage I through III CRC which is contrary to current guidelines.

## Declarations

### Author contribution statement

Alissa Greenbaum: Conceived and designed the experiments; Performed the experiments; Analyzed and interpreted the data; Wrote the paper.

Manuel Rojo: Conceived and designed the experiments; Analyzed and interpreted the data; Wrote the paper.

Charles Wiggins, Angela Meisner, Anita Kinney, Ashwani Rajput: Conceived and designed the experiments; Performed the experiments; Analyzed and interpreted the data; Contributed reagents, materials, analysis tools or data; Wrote the paper.

### Funding statement

This work was supported by the University of New Mexico Department of Surgery. This project was supported, in part, by National Cancer Institute ContractHHSN2612001300010, Task Order HHSN26100005. Additional support was provided by the National Cancer Institute and the University of New Mexico Comprehensive Cancer Center Support Grant through Grant Number 2P30CA118100-11.

### Competing interest statement

The authors declare no conflict of interest.

### Additional information

No additional information is available for this paper.

## References

[1] Siegel R.L., Miller K.D., Jemal A. (2016). Cancer statistics. CA Cancer J. Clin..

[2] Calonge N., Fisher N.L., Berg A.O. (2013). Recommendations from the EGAPP Working Group: can testing of tumor tissue for mutations in EGFR pathway downstream effector genes in patients with metastatic colorectal cancer improve health outcomes by guiding decisions regarding anti-EGFR therapy. Genet. Med..

[3] Allegra C.J., Jessup J.M., Somerfield M.R. (2009). American Society of Clinical Oncology provisional clinical opinion: testing for KRAS gene mutations in patients with metastatic colorectal carcinoma to predict response to anti-epidermal growth factor receptor monoclonal antibody therapy. J. Clin. Oncol..

[4] Bos J.L., Fearon E.R., Hamilton S.R. (1987). Prevalence of ras gene mutations in human colorectal cancers. Nature.

[5] Peeters M., Kafatos G., Taylor A. (2015). Prevalence of RAS mutations and individual variation patterns among patients with metastatic colorectal cancer: A pooled analysis of randomised controlled trials. Eur. J. Cancer.

[6] Webster J., Kauffman T.L., Feigelson H.S. (2013). KRAS testing and epidermal growth factor receptor inhibitor treatment for colorectal cancer in community. Cancer Epidemiol. Biomark Prev..

[7] Allegra C.J., Rumble R.B., Hamilton S.R. (2016). Extended RAS Gene Mutation Testing in Metastatic Colorectal Carcinoma to Predict Response to Anti-Epidermal Growth Factor Receptor Monoclonal Antibody Therapy: American Society of Clinical Oncology Provisional Clinical Opinion Update 2015. J. Clin. Oncol..

[8] Bokemeyer C., Köhne C.H., Ciardiello F., Lenz H.J., Heinemann V., Klinkhardt (2015). FOLFOX4 plus cetuximab treatment and RAS mutations in colorectal cancer. Eur. J. Cancer.

[9] Bokemeyer C., Bondarenko I., Hartmann J.T. (2011). Efficacy according to biomarker status of cetuximab plus FOLFOX-4 as first-line treatment for metastatic colorectal cancer: the OPUS study. Ann. Oncol..

[10] De Roock W., Piessevaux H., De Schutter J. (2008). KRAS wild-type state predicts survival and is associated to early radiological response in metastatic colorectal cancer treated with cetuximab. Ann. Oncol..

[11] Van Cutsem E., Köhne C.H., Láng I. (2011). Cetuximab plus irinotecan, fluorouracil, and leucovorin as first-line treatment for metastatic colorectal cancer: updated analysis of overall survival according to tumor KRAS and BRAF mutation status. J. Clin. Oncol..

[12] Douillard J.Y., Siena S., Cassidy J. (2014). Final results from PRIME: randomized phase III study of panitumumab with FOLFOX4 for first-line treatment of metastatic colorectal cancer. Ann. Oncol..

[13] Lin A.Y., Buckley N.S., Lu A.T., Kouzminova N.H., Salpeter S.R. (2011). Effect of KRAS mutational status in advanced colorectal cancer on the outcomes of anti-epidermal growth factor receptor monoclonal antibody therapy: a systematic review and meta-analysis. Clin. Colorectal Cancer.

[14] Yen L.C., Uen Y.H., Wu D.C. (2010). Activating KRAS mutations and overexpression of epidermal growth factor receptor as independent predictors in metastatic colorectal cancer patients treated with cetuximab. Ann. Surg..

[15] Trojan J., Mineur L., Tomasek J. (2015). Panitumumab use in metastatic colorectal cancer and patterns of KRAS testing: results from a Europe-wide physician survey and medical records review. PLos One.

[16] Carter G.C., Landsman-Blumberg P.B., Johnson B.H. (2015). KRAS testing of patients with metastatic colorectal cancer in a community-based oncology setting: a retrospective database analysis. J. Exp. Clin. Cancer Res..

[17] Ciardiello F., Tejpar S., Normanno N. (2011). Uptake of KRAS mutation testing in patients with metastatic colorectal cancer in Europe, Latin America and Asia. Target Oncol..

[18] Charlton M.E., Karlitz J.J., Schlichting J.A. (2017). Factors associated with guideline-recommended KRAS testing in colorectal cancer patients: A Population-based Study. Am. J. Clin. Oncol..

[19] Ward M., Ulrich F., Matthews K. (2014). Access to chemotherapy services by availability of local and visiting oncologists. J. Clin. Oncol. Practice.

[20] Baldwin L., Andrilla C., Porter M., Rosenblatt R.A., Patel S., Doescher M.P. (2013). Treatment of early-stage prostate cancer among rural and urban patients. Cancer.

[21] Provenzale D., Jasperson K., Ahnen D.J. (2015). Colorectal Cancer Screening. Version 1.2015. J. Natl. Compr. Canc. Netw..

[22] Zeng C., Wen W., Morgans A.K., Pao W., Shu X.O., Zheng W. (2015). Disparities by race, age, and sex in the improvement of survival for major cancers: results from the National Cancer Institute Surveillance, Epidemiology, and End Results (SEER) Program in the United States, 1990 to 2010. JAMA Oncol..

[23] Rodriguez R., Gonzales M., Fahy B., Kinney A., Hoffman R., Rajput A. (2015). Disparities in stage at presentation and treatment of colorectal cancer among Hispanic and non-Hispanic White patients. Am. Surg..

[24] Edge S.B., Byrd D.R., Compton C.C., Fritz A.G., Greene F.L., Trotti A. (2010). AJCC cancer staging manual.

[25] National Comprehensive Cancer Network (2017). Colon Cancer (Version 1). https://www.nccn.org/professionals/physician_gls/pdf/colon.pdf.

[26] Kadowaki S., Kakuta M., Takahashi S. (2015). Prognostic value of KRAS and BRAF mutations in curatively resected colorectal cancer. World J. Gastroenterol..

[27] Lee D.W., Kim K.J., Han S.W. (2015). KRAS mutation is associated with worse prognosis in stage III or high-risk stage II colon cancer patients treated with adjuvant FOLFOX. Ann. Surg. Oncol..

[28] Lin C.C., Bruinooge S.S., Kirkwood M.K. (2015). Association between geographic access to cancer care, insurance, and receipt of chemotherapy: geographic distribution of oncologists and travel distance. J. Clin. Oncol..

[29] Erickson C., Salsberg E., Forte G., Bruinooge S., Goldstein M. (2007). Future supply and demand for oncologists: challenges to assuring access to oncology services. J. Oncol. Pract..

[30] Hines R., Markossian T., Johnson A., Dong F., Bayakly R. (2013). Geographic residency status and census tract socioeconomic status as determinants of colorectal cancer outcomes. Am. J. Public Health.

[31] Singh G.K., Williams S.D., Siahpush M. (2011). Socioeconomic, rural-urban, and racial inequalities in U.S. cancer mortality: Part I- all cancers and lung cancer and Part II- colorectal, prostate, breast and cervical cancers. J. Cancer Epidemiol..

[32] Chow C.J., Al-Refaie W.B., Abraham A. (2015). Does patient rurality predict quality colon cancer care? A population-based study. Dis. Colon Rectum..

[33] Sparling A.S., Song E., Klepin H.D., Foley K.L. (2016). Is distance to chemotherapy an obstacle to adjuvant care among the N.C. Medicaid-enrolled colon cancer patients?. J. Gastrointest. Oncol..

[34] Kurniali P.C., Hrinczenko B., Al-Janadi A. (2014). Management of locally advanced and metastatic colon cancer in elderly patients. World J. Gastroenterol..

[35] Schiphorst A.H.W., Verweji N.M., Pronk A., Hamaker M.E. (2014). Age-related guideline adherence and outcome in low rectal cancer. Dis. Colon Rectum..

[36] Foster J.A., Salinas G.D., Mansell D., Williamson J.C., Casebeer L.L. (2010). How does older age influence oncologists’ cancer management?. Oncologist.

[37] Haris J.N., Liljestrand P., Alexander G.L. (2013). Oncologist’s attitudes toward KRAS testing: a multisite study. Cancer Med..

[38] Smith T.J., Hillner B.E. (2001). Ensuring quality cancer by the use of clinical practice guidelines and critical pathways. J. Clin. Oncol..

[39] Deng Y., Wang L., Tan S. (2015). KRAS as a predictor of poor prognosis and benefit from postoperative FOLFOX chemotherapy in patients with stage II and III colorectal cancer. Mol. Oncol..

[40] Roth A.D., Tejpar S., Delorenzi M. (2010). Prognostic role of KRAS and BRAF in stage II and III resected colon cancer: results of the translational study on the PETACC-3, EORTC 40993, SAKK 60-00 trial. J. Clin. Oncol..

